# Impact of modified techniques on outcomes of peroral endoscopic myotomy: A narrative review

**DOI:** 10.3389/fmed.2022.948299

**Published:** 2022-08-18

**Authors:** Zaheer Nabi, D. Nageshwar Reddy

**Affiliations:** Asian institute of Gastroenterology, Hyderabad, India

**Keywords:** per-oral endoscopic myotomy (POEM), achalasia, outcomes, gastroesophageal reflux, complication, clinical success

## Abstract

Peroral endoscopic myotomy (POEM) is an established frontline treatment modality for achalasia cardia. Since its initial description, several modifications have been proposed to the technique of POEM. Broadly speaking, these modifications follow the basic principles of submucosal endoscopy, but incorporate variations in the POEM technique, including the difference in the orientation of myotomy (anterior or posterior), length of myotomy (short or long), and thickness of myotomy (selective circular or full thickness). Some of these modifications have been shown to reduce procedural duration without compromising the efficacy of the POEM procedure. More recently, several alterations have been reported that intend to reduce gastroesophageal reflux after POEM. These include preservation of sling fibers during posterior POEM and addition of NOTES fundoplication to the POEM procedure. Although some of the modified techniques have been compared with the conventional techniques in quality trials, randomized studies are awaited for others. The incorporation of some of these modifications will likely make POEM a technically easy and safer modality in near future. This review aims to discuss the current evidence with regard to the impact of modified techniques on the outcome of POEM.

## Introduction

Peroral endoscopic myotomy (POEM) has emerged as a safe and effective modality for the management of achalasia, as well as non-achalasia esophageal motility disorders (1). The first series of POEM was published over a decade ago (2). Since then multiple studies have confirmed the utility of POEM in esophageal motility disorders. The updated guidelines by major gastrointestinal societies have acknowledged the role of POEM as one of the frontline treatment modalities for the management of achalasia (3–6).

POEM is a technique in evolution with new information being generated at a fairly constant pace. The standard technique of POEM includes 8–10 cm of myotomy *via* an anterior or posterior route (2, 7). Several technical modifications and variations in the technique of POEM have been evaluated and compared with the standard technique. These modifications include orientation of myotomy, length of myotomy, thickness of myotomy, and antireflux myotomy. This narrative review focuses on the impact of different techniques on the outcomes of POEM.

## Orientation of myotomy: Anterior, posterior, and greater curve

The orientation of myotomy constitutes the most common variation in the technique of POEM. The initial description of POEM was through the anterior (1–2 o’clock) route (2). Subsequently, the safety and feasibility of POEM *via* the posterior route (5 o’clock) were reported.

Several randomized controlled trials have compared the outcomes of POEM when performed *via* an anterior or posterior approach (8–10). The clinical success has been found to be comparable between the two orientations of myotomy in all the trials at 6–12 months follow-up period. More recently, Ichkhanian et al. confirmed the comparable efficacy of anterior and posterior myotomy at 2-year follow-up (anterior 85% vs. posterior 79%) (11).

With regard to the adverse events, the risk of mucosal injuries may be higher after anterior POEM (8). During anterior myotomy, acute tip angulation is required to hook the circular muscle layer, which causes greater “fling” of the knife predisposing to mucosal injuries (12). On the other hand, less tip angulation is required during posterior myotomy as the electrosurgical knife emerges from 5 to 6 o’clock. Therefore, POEM may be technically easier with a shorter procedure duration when performed by the posterior route (13).

Posterior POEM involves severance of both the circular and sling or oblique fibers potentially leading to a higher incidence of gastroesophageal reflux disease (GERD). Abnormal esophageal acid exposure was higher in the posterior myotomy group in one randomized study and similar in the other two randomized controlled trials (8–10). In a recent systematic review and meta-analysis, circular and anterior myotomy demonstrated a lower trend of GERD with borderline significance (14).

In some cases, myotomy by conventional routes (anterior or posterior) may not be feasible due to anatomical reasons (esophageal diverticula, disease progression) or submucosal fibrosis (SMF) as a result of previous treatments (endoscopic submucosal dissection or prior POEM). In these cases, endoscopic myotomy can be performed successfully *via* a greater curvature approach, i.e., 8 o’clock. In a prospective study, including 21 cases, Onimaru et al. reported the feasibility and efficacy of greater curvature myotomy (15). All the cases were successfully performed and clinical success was recorded in 95.2% of cases. Post-POEM reflux esophagitis (majority grades A and B) was seen in almost half of the cases. The downside of greater curvature myotomy is a relatively high incidence of GERD due to the inclusion of angle of His while performing myotomy.

## Thickness of myotomy: Complete vs. selective circular

Circular muscles are mainly responsible for the genesis of symptoms in patients with achalasia and the role of longitudinal muscle remains debatable. Some experts have highlighted the disco-ordination between longitudinal and circular muscles in patients with achalasia and the role of longitudinal muscles in the generation of symptoms, such as dysphagia and chest pain (16). In the initial studies, a selective circular myotomy was described (2). Subsequent studies described a progressive full-thickness myotomy (selective circular in upper portion and full thickness in distal) and complete full-thickness myotomy during the POEM procedure. Limited data comparing the outcomes between full-thickness and selective circular myotomy suggest that besides reduced procedure duration with full-thickness myotomy, there is no clinically relevant difference between the two techniques, especially with regard to clinical success (17–19). The incidence of GERD was higher after full-thickness myotomy in one study and similar in another study (17, 19). Randomized controlled trials with adequate follow-up are required in the future to determine the impact of full-thickness myotomy on the incidence of post-POEM reflux.

## Length of myotomy: Short vs. standard

The length of myotomy in the initial study reported by Inoue et al. was 8.1 cm (esophagus 6.1 cm and gastric 2 cm) (2). Subsequent studies described more or less similar length of myotomies. Although the length of gastric myotomy has been shown to impact clinical success after surgical myotomy, the same may not be true for esophageal myotomy (20). Recent studies have challenged the “dogma” of long esophageal myotomies. Wang et al. initially described good outcomes of short myotomy (5.4 cm) in 46 patients with achalasia (21). However, short follow-up (3 months) and lack of a comparison arm were the main limitations. Subsequently, one retrospective comparative study and two randomized controlled trials have confirmed that a short myotomy is equally efficacious to standard myotomy in cases with type I and II achalasia (22–24). In a randomized study, including 71 patients, Nabi et al. compared the outcomes between short (≤3 cm) and standard (≥6 cm) esophageal myotomies in cases with type I and II achalasia (24). The mean length of esophageal myotomy was 2.76 ± 0.41 cm in the short and 7.97 ± 2.40 cm in the long myotomy groups, respectively. The mean operating duration was significantly less in the short esophageal myotomy group (44.03 ± 13.78 min vs. 72.43 ± 27.28 min, *P* < 0.001). At 1 year, clinical success (Eckardt score ≤ 3) was similar in both the groups (short 93.6% vs. long 96.9%) (24). Similar conclusions were drawn in another randomized study from China, which included treatment-naïve patients with type II achalasia (23). However, in contrast to the study by Nabi et al. postoperative abnormal esophageal acid exposure occurred more often in the standard myotomy group than in the short myotomy group (43.8% vs. 23.9%, *p* = 0.042). In a systematic review and meta-analysis, including five studies (521 patients), short and standard myotomies were similar with respect to clinical success, hospital stay, reflux esophagitis, and adverse events (25). However, abnormal esophageal acid exposure was less frequent and the procedure duration significantly shorter in the short myotomy group.

In conclusion, short esophageal myotomy is associated with reduced procedure duration without compromising the efficacy for at least 1 year after the POEM procedure. The literature is divergent with regard to the impact of short myotomy on the prevention of post-POEM gastroesophageal reflux and further studies are required.

## Anti-reflux peroral endoscopic myotomy

GERD is the most common adverse event after POEM in long term. Although symptomatic reflux is uncommon, erosive esophagitis and reflux by pH monitoring are detected in up to two-thirds of patients after POEM (26, 27). In randomized controlled trials, GERD was more common after POEM as compared to pneumatic dilation and Heller myotomy with fundoplication (28, 29). The fact that there is a poor correlation between symptoms and increased esophageal acid exposure indicates that GERD should be searched proactively after POEM. In addition, novel techniques need to be devised to prevent GERD after POEM.

In this regard, preservation of sling fibers during posterior POEM and NOTES fundoplication has emerged as potential strategies to prevent reflux after POEM. Sling or oblique fibers appear along the gastric side during posterior POEM and form one of the important components of the natural antireflux mechanism. Therefore, it appears logical that preservation of sling fibers during myotomy along the gastric side may reduce the incidence of GERD after POEM. Tanaka et al. compared the incidence of reflux esophagitis in 114 patients who underwent POEM either by conventional technique (31 cases) or sling fiber preservation technique (83 cases) (30). The incidence of ≥ grade B esophagitis was significantly lower in the cases where sling fibers were preserved (31.3% vs. 58.1%, *p* = 0.017). However, symptomatic reflux was not significantly different in the two groups (10.8% vs. 19.4%, *p* = 0.23). Of note, the second penetrating vessel has also been shown to be a reliable landmark for the distal end of POEM (31). Penetrating vessels form the boundary between the circular (right of vessels) and the oblique fibers (left of vessels). Therefore, performing gastric myotomy toward the right of penetrating vessels preserves the sling or oblique fibers during POEM (32).

Other techniques to prevent reflux after POEM include avoiding excess gastric myotomy (>4 cm) to prevent severing of oblique fibers, short esophageal myotomy, and selective circular myotomy during POEM (17, 23, 33). Grimes et al. evaluated the impact of gastric length of myotomy on the incidence of post-POEM reflux esophagitis (33). The authors used the double-scope technique to gauge the extent of gastric myotomy in one group. The mean length of myotomy was significantly longer in the double-scope group (3.3 cm vs. 2.6 cm). Although the overall incidence of reflux esophagitis was similar in the two groups, moderate esophagitis (LA grade B) was significantly higher in the double-scope group (25% vs. 4%). The authors concluded that gastric myotomy length affects severity, but not the rate of postprocedure reflux.

One of the reasons for a higher rate of reflux after POEM is that an antireflux procedure is not performed. In a proof of concept study, Inoue et al. performed endoscopic fundoplication in 21 patients who underwent POEM *via* anterior route (34). The fundoplication procedure involved entry into the peritoneum and creating a wrap by approximating the serosal aspect of the anterior gastric wall toward the distal end of myotomy using an endoloop and multiple endoclips. Bapaye et al. reported the 1-year follow-up data in twenty-five patients who underwent NOTES fundoplication (35). The fundoplication wrap was intact in the majority (82.6%) of the patients and abnormal esophageal acid exposure was detected in only two patients. Reflux esophagitis was seen in 18.2% and all of them had mild esophagitis (grade A). Although the preliminary results are encouraging, concerns have been raised regarding the durability of the results and leaving foreign bodies *in situ*. Moreover, the published studies suggest a progressive increase in reflux after Heller myotomy with fundoplication on long-term follow-up (36). Therefore, it is likely that the wrap created during POEM-F may deteriorate and lead to higher rates of reflux with time. More recently, Toshimori et al. described a refined technique of POEM-F where they used endoscopic hand suturing (instead of endoloop and clips) to create a fundoplication wrap (37). Larger trials and long-term follow-up data are required before incorporating NOTES fundoplication into routine clinical practice.

Wang et al. compared the incidence of clinically relevant GERD (abnormal esophageal acid exposure with reflux esophagitis or symptoms) in 56 patients who underwent circular or full-thickness myotomy (17). Clinically relevant GERD was significantly higher in the full-thickness myotomy group (37.5% vs. 12.5%; *p* < 0.05). In another study (34 patients), the incidence of symptomatic GERD and reflux esophagitis was similar in the partial full-thickness myotomy and the full-thickness myotomy groups (18). Contrasting results from these studies suggest that randomized controlled trials comparing circular myotomy to full-thickness myotomy are required before concluding the benefits of selective circular or partial-thickness myotomy for the prevention of GERD.

## Technique of D-peroral endoscopic myotomy: Septotomy vs. no septotomy

Epiphrenic diverticula of the esophagus (EED) is a rare pulsion-type diverticulum that forms due to increased intraesophageal pressure resulting in herniation of mucosal and submucosal layers (38). Submucosal tunneling (D-POEM) is emerging as a safe and effective treatment in cases with EED. The conventional technique of D-POEM involves submucosal tunneling, exposure of septum, and, finally, division of septum (39). In addition, myotomy of the lower esophageal sphincter is also performed as the majority of the cases have coexistent esophageal motility disorders. With this approach, Nabi et al. reported a clinical success of 84.6% at a median follow-up of 25 months (38). More recently, the utility of septotomy has been questioned, especially in cases with associated esophageal motility disorders (40–42). Kinoshita et al. evaluated the efficacy of POEM without septotomy in fourteen cases with EED (41). The median size of the diverticulum was 29 mm (9–90). There was a significant improvement in symptoms, as well as integrated relaxation pressures in all the patients at 3-month follow-up. The authors concluded that POEM alone is effective for patients with esophageal motility disorders and EED. Similar conclusions were drawn in another study suggesting that a decrease of the lower esophageal sphincter relaxation pressure even without diverticulotomy may be an effective alternative to D-POEM (40). Of note, the follow-up period was short (3 months) in both of these studies. Moreover, there is no randomized or non-randomized study comparing these two approaches. Therefore, comparative studies with adequate follow-up duration are required in the future. In a recent review, Samanta et al. proposed a personalized approach to EED (43). The authors suggested that POEM alone may be sufficient in cases with small EED and coexistent esophageal motility disorders. However, septotomy is required in cases with large EED and those without an evident motility disorder. In the absence of quality evidence, the utility of such an approach is subjected to future studies.

## Other modifications

Other modifications in the technique of POEM that have been described include modified techniques of mucosal incision, double tunnel technique and simultaneous tunneling and myotomy in cases with severe submucosal fibrosis, use of guidewire, and double-scope technique in cases with advanced sigmoid-type achalasia.

## Mucosal incision techniques

In the vast majority of studies, a longitudinal mucosal incision is utilized to gain entry into the submucosal tunnel. The main advantage of a longitudinal incision is easy closure. The potential downsides include difficult entry into the tunnel and a higher risk of insufflation-related adverse events as the endoscope snugly fits the incision. Zhai et al. reported the utility of a transverse incision during POEM (44). The incidence of insufflation-related adverse events was significantly lower in the transverse incision group when compared to cases with longitudinal entry incision (9.8% vs. 41.7%). The authors concluded that POEM with a transverse entry incision can significantly decrease the operation time and reduce the incidence of pneumatosis-related complications (44). The proposed advantages of transverse mucosal incision include easier entry into the tunnel, while facilitating the egress of gas from the tunnel. An obvious downside of transverse incision is difficulty in the closure using endoclips. Ma et al. described an inverted “T”-shaped incision for entry into the tunnel and concluded its role in reducing complications associated with POEM (45). A recent consensus statement on the digestive endoscopic tunnel technique recommended an inverted “T” mucosal incision based on the advantages, including easier entry, low gas-related events, and requirements of fewer clips for closure (level of evidence: III; strength of recommendation: B). While the modified mucosal incision techniques appear advantageous, there is no randomized controlled trial demonstrating their superiority over the longitudinal incision. Besides, presumed difficulty in the closure is another reason that modified incision techniques have not gained widespread acceptance.

## Peroral endoscopic myotomy in difficult cases

The presence of submucosal fibrosis (SMF) may pose special challenges to the POEM procedure. In some studies, severe SMF has been found to be the most important reason for technical failure during POEM. In these cases, modified techniques may overcome the technical difficulties during POEM. Liu et al. evaluated the outcomes of a modified technique of POEM (open-POEM) in 82 patients with achalasia (46). In this technique, a mucosal incision was created 6–10 cm above the gastroesophageal junction and extended at least 2 cm beyond the gastroesophageal junction. Subsequently, submucosal dissection and selective circular myotomy were performed without creating a submucosal tunnel. Technical success was achieved in all the patients and the mean procedure duration was 20 min. At a median follow-up of 18 months, clinical success was recorded in 96.3% of patients. The authors proposed that open POEM may be advantageous with regard to short procedure duration and low rate of insufflation-related adverse events. In addition, open POEM may be useful in cases with SMF where the creation of submucosal tunnel may be especially difficult. While acknowledging the potential advantages, it is prudent to realize the possible limitations of this technique. First, the risk of perforation may be especially high as the mucosal flap is not intact and the thin layer of longitudinal muscle fibers may split easily. The fact that three cases developed mediastinitis after open POEM suggests that caution is advised while adopting O-POEM in routine clinical practice. Besides O-POEM, another technique that potentially circumvents the issue of SMF is the “double tunnel technique” (47, 48). We reported the utility of this technique in 11 cases of achalasia with severe SMF (48). In brief, the technique involves the creation of a second submucosal tunnel along different orientations in the esophagus after technical failure with the first tunnel due to severe SMF. In our experience, SMF is usually focal and POEM can be successfully performed *via* the second submucosal tunnel.

In cases with advanced sigmoid achalasia, it may be difficult to maintain orientation during submucosal tunneling mainly attributable to the tortuosity of the esophagus. Several techniques have been reported to circumvent the issue of losing orientation during POEM and including fluoroscopy-guided POEM, use of guidewire, double-scope method, and open POEM technique (49–51). These techniques have been described in case reports and small case series and, therefore, utilized as a “last-ditch effort” in difficult POEM cases.

## Summary

POEM is an established treatment modality for esophageal motility disorders. Nevertheless, unlike pneumatic dilatation and Heller myotomy, POEM is a relatively new tool in the armamentarium for the management of achalasia. Several different techniques of POEM have evolved over the last decade. These modifications intend to simplify the POEM procedure either by reducing procedure duration (short myotomy, full-thickness myotomy), improving technical success in challenging cases (double-tunnel technique), or reducing reflux after POEM (sling fiber preservation, NOTES fundoplication) (Table 1). Some of the techniques have undergone rigorous comparison in randomized controlled trials (anterior vs. posterior POEM and short vs. long myotomy). On the other hand, the impact of other modifications (full thickness, open POEM) is subjected to quality trials in the future. More recently, the focus of studies has shifted from clinical efficacy to prevention of GERD after POEM. Although the outcomes of antireflux POEM techniques appear promising, robust data are yet to make an appearance. Nevertheless, the time has come to formulate the best combination for optimal outcomes. For example, a short, anterior, or sling fiber-preserving posterior circular myotomy may shorten the procedure duration and reduce postoperative GERD without compromising the efficacy of POEM. Needless to say that not all the modifications will sustain and some may perish for lack of clinical relevance.

**TABLE 1:** Modified techniques of peroral endoscopic myotomy: Current status and future directions.

		Technique	Current evidence	Future directions
1.	Orientation of myotomy	Anterior vs. posterior POEM	Clinical success and GERD similar at 1 year (RCTs +)	Long-term follow-up studies
2.	Thickness of myotomy	Selective circular vs. full thickness myotomy	Clinical success similar, GERD may be similar or higher after full thickness myotomy (No RCTs)	Randomized comparison studies, impact on GERD needs to further evaluation
3.	Length of myotomy	Short vs. standard myotomy	Clinical success similar at 1 year, GERD may be similar or higher after long myotomy (RCTs +)	Long-term follow-up studies required to confirm the durability of response to short myotomy
4.	Diverticular POEM	Septotomy vs. no septotomy	POEM alone may be sufficient and septotomy may not be required (No RCTs)	Long term results of POEM without septotomy, comparative studies between the two techniques
5.	Anti-reflux POEM	Sling fiber preservation, NOTES-fundoplication	Both techniques may potentially prevent post POEM reflux (No RCTs)	Quality studies required to confirm the utility of anti-reflux POEM techniques
6.	Submucosal fibrosis	Open-POEM, double tunnel POEM	Both techniques appear be useful in cases with severe SMF (No RCTs)	Safety of O-POEM needs evaluation in future studies

*POEM, per-oral endoscopic myotomy; GERD, gastroesophageal reflux disease; RCTs, randomized controlled trials.*

## Conclusion

POEM is a frontline treatment modality for achalasia cardia along with pneumatic dilatation and Heller myotomy. The orientation of myotomy and the length of esophageal myotomy do not impact the outcomes of POEM. Several modifications, including selective circular myotomy, preservation of sling fibers, and NOTES fundoplication, may reduce the incidence of GERD after POEM. However, quality data are lacking and further studies are required.

## Author contributions

ZN involved in literature review and drafting the manuscript. DN provided critical inputs to the manuscript. Both authors agreed to the final version of the manuscript.
